# Advancements in Ocular Regenerative Therapies

**DOI:** 10.3390/biology12050737

**Published:** 2023-05-19

**Authors:** Wojciech Tomczak, Weronika Winkler-Lach, Martyna Tomczyk-Socha, Marta Misiuk-Hojło

**Affiliations:** 1 Lower Silesian Oncology Center, 53413 Wroclaw, Poland; 2 Department of Ophthalmology, Wroclaw Medical University, 50556 Wroclaw, Poland

**Keywords:** regenerative medicine, stem cell therapy, limbal transplantation

## Abstract

**Simple Summary:**

In recent years, ophthalmology has witnessed groundbreaking advancements in the utilisation of stem cells to revolutionise the treatment of various ocular disorders. Stem cells, with their unique ability to differentiate into specialised cell types, hold immense potential for restoring vision and healing damaged tissues. The ability to stop or even reverse vision loss through stem cell therapies could significantly enhance patients’ quality of life and reduce the burden on healthcare systems. In addition, the ocular accessibility, availability of non-invasive follow-up, and immunological privilege of the eye tissues are driving the development of SC use in ophthalmology. We believe that regular reports on the proven applications of stem cells will certainly sooner or later change the patterns of conduct in today’s ophthalmology. This study serves to provide a comprehensive summary of the latest advancements in the utilisation of stem cells in the field of ophthalmology.

**Abstract:**

The use of stem cells (SCs) has emerged as a promising avenue in ophthalmology, offering potential therapeutic solutions for various vision impairments and degenerative eye diseases. SCs possess the unique ability to self-renew and differentiate into specialised cell types, making them valuable tools for repairing damaged tissues and restoring visual function. Stem cell-based therapies hold significant potential for addressing conditions such as age-related macular degeneration (AMD), retinitis pigmentosa (RP), corneal disorders, and optic nerve damage. Therefore, researchers have explored different sources of stem cells, including embryonic stem cells (ESC), induced pluripotent stem cells (iPSCs), and adult stem cells, for ocular tissue regeneration. Preclinical studies and early-phase clinical trials have demonstrated promising outcomes, with some patients experiencing improved vision following stem cell-based interventions. However, several challenges remain, including optimising the differentiation protocols, ensuring transplanted cells’ safety and long-term viability, and developing effective delivery methods. The field of stem cell research in ophthalmology witnesses a constant influx of new reports and discoveries. To effectively navigate these tons of information, it becomes crucial to summarise and systematise these findings periodically. In light of recent discoveries, this paper demonstrates the potential applications of stem cells in ophthalmology, focusing on their use in various eye tissues, including the cornea, retina, conjunctiva, iris, trabecular meshwork, lens, ciliary body, sclera, and orbital fat.

## 1. Introduction

**Figure 1:**
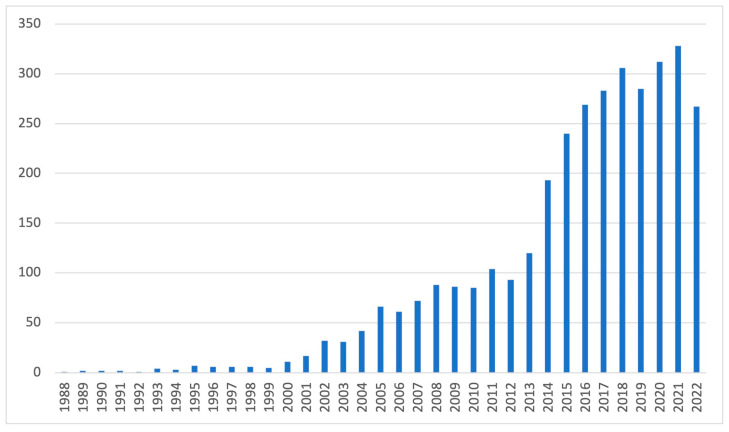
Number of papers on stem cells in ophthalmology depending on the year of publication in PubMed database.

The term stem cell (SC) was first introduced in 1868 [1,2]. We define them as ones from which other, more specialised cell types develop whilst maintaining their population. They can be divided into pluripotent stem cells, including embryonic and induced pluripotent stem cells, and non-embryonic somatic stem cells, also known as adult SCs. Pluripotent SCs can transform into each cell of an adult organism compared to adult, which can differentiate into specialised cells of the tissues in which they are located [3]. Since discovering the aforementioned stem cell capabilities, the scientific community has sought applications for the newly established regenerative medicine sector. Gradually, our knowledge has expanded to the point that it now guides the development of the majority of specialties. For example, the number of published papers on stem cells in 2020 exceeded the cumulative total until the end of 1990 according to the PubMed database. Figure 1. expresses increasing interest in SCs as measured by the quantity of published research in PubMed in the field of ophthalmology over time.

Nowadays, cell therapy is a prominent intervention that is being developed or used in a variety of medical conditions, including lung [4,5,6,7,8], cardiovascular [9,10,11], liver [12,13], and kidney conditions [14,15]. In addition, recent studies have demonstrated that mesenchymal stem cells (MSCs) can treat COVID-19 patients’ pulmonary fibrosis, improve lung function, and reduce inflammation [16,17]. Further, SCs may be a promising therapy for Alzheimer’s disease, whereas genetically altered SCs can enhance brain function and reduce degenerative characteristics [18,19,20]. After animal models showed positive results, Alzheimer’s patients started participating in new clinical trials [18,19,20].

The following noteworthy approach combines SCs with nanotechnology, potentially opening new SC production and research strategies. This combined approach potential uses are imaging and labelling, drug or gene delivery, tissue scaffold engineering, and monitoring of stem cell proliferation.

The application of SCs in ophthalmology has been driven by our understanding of the genetic and environmental factors contributing to eye diseases and how vision loss impacts cognitive and psychosocial health [21]. In addition, the ocular accessibility, availability of non-invasive follow-up, and immunological privilege of the eye tissues all contribute to the effectiveness of applying SCs in ophthalmology.

**Figure 2:**
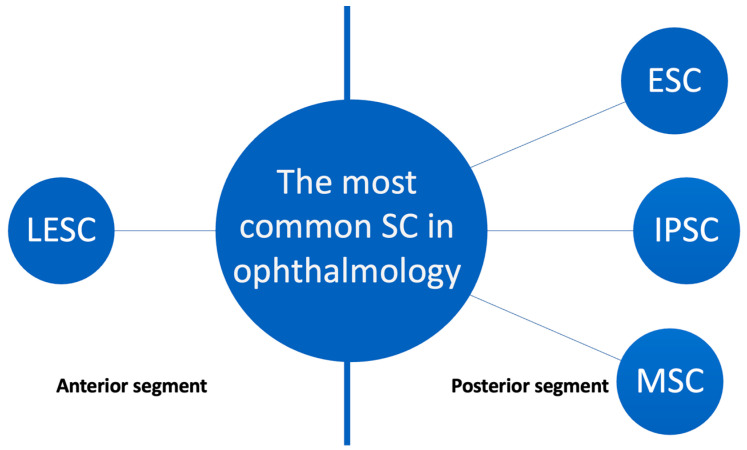
The most common SC used in ophthalmology (LESC—limbal epithelial stem cell, ESC—embryonic stem cell, iPSC—induced pluripotent stem cell, MSC—mesenchymal stem cell).

Currently, SCs are used in ophthalmology, yet mostly in clinical trials. SCs are mainly used in diseases of the cornea and conjunctiva, but more applications of stem cell transplants to various eye tissues are described. Along with searching for novel SC applications, researchers also look for new sources and types of SCs. The most used SCs are shown in Figure 2.

Limbal Epithelial Stem Cells (LESCs) are primarily located in the corneal limbus, specifically along the palisades of Vogt. These cells play a crucial role in the continuous renewal of the cornea. Moreover, they are essential in repairing the corneal surface after several damage. LESC and mesenchymal stem cells (MSCs) are the only SCs that have been successfully used in clinical settings so far. Unlike human embryonic stem cells (hESCs), which raise legal and ethical concerns due to their extraction from blastocysts, MSCs obtained from adult tissues present fewer issues. MSCs are stromal cells capable of self-renewal and differentiation into various lineages. Additionally, they can be derived from diverse sources such as the umbilical cord, endometrial polyps, menstrual blood, bone marrow, and adipose tissue.

At the time of writing, nine clinical trials are underway to prove the effectiveness of stem cell therapy in diseases currently considered incurable, such as Stargard’s disease and other retinal diseases and optic nerve neuropathies [22]. A comprehensive summary of these nine clinical trials can be found in Table S1.

**Figure 3:**
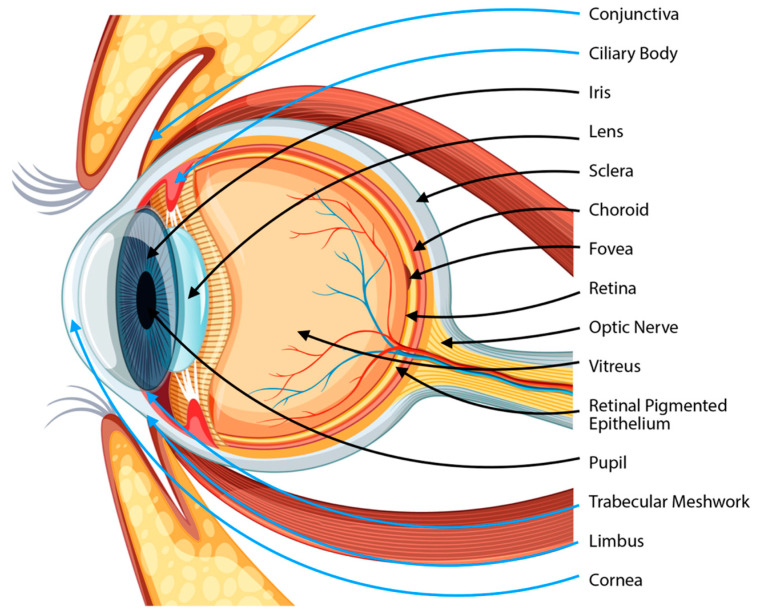
The anatomy of the eye, considering the location of stem cells (blue arrows).

The growing use of SCs will be outlined below by particular eye tissue. The most crucial eyeball anatomical components are shown in Figure 3; blue arrows indicate the locations of the stem cell-containing structures. Adapted from https://www.freepik.com/free-vector/diagram-human-eyeball-anatomy_13832801.htm (accessed on 6 March 2023).

## 2. Cornea

### 2.1. Basic Knowledge

The cornea is the outermost component of the eye’s optical system with the greatest focusing power. It comprises five layers, each of which plays a crucial role in maintaining its transparency [21]. The outermost epithelium comprises five to seven layers of cells, with its turnover time estimated at around four days. This epithelium is embedded in the basal membrane called Bowman’s. The stroma constitutes up to 90% of the thickness of the cornea and is mainly composed of numerous layers of parallel collagen fibres that cross each other at right angles. Its posterior surface is covered with a Descemet membrane on which a single-layer flat corneal endothelium rests. Endothelium cells are responsible for the hydration of the stroma and do not proliferate, which makes them very susceptible to damage. In such a situation, the remaining cells compensate for the defects by enlarging and stretching to maintain the layer’s continuity. In addition, the cornea absorbs nutrients from the aqueous humor and oxygen via the tear film. A sixth, situated between the Descemet membrane’s matrix and membrane, was presented in 2013 [23].

According to the estimates, corneal opacities, currently the fourth most common cause of visual impairment, result in moderate to severe distant vision loss or blindness in 4.2 million individuals globally [24]. Therefore, identifying corneal epithelial stem cells in its limbus (LESC) was one of the first significant discoveries for the progress of stem cell therapy and opened the door to new treatment methods, not only for patients treated in the ophthalmology department [25]. LESCs are abundant along the superior and inferior limbus Vogt palisades, which are radially oriented fibrovascular ridges [26].

The limbus acts as a protective environment and supports their self-renewal. Injuries but also congenital and acquired diseases can lead to limbal stem cell deficiency (LSCD). Chemical and thermal burns account for 75% of all LSCD cases [27,28]. Lack of LESC eventually causes conjunctiva invasion of the cornea, neovascularisation, and visual clouding and degradation [29]. Stem cells give people a chance to regain transparent corneas without surgery. Although the described sequence of events is evident, we are still looking for different ways to stop or reverse these changes.

### 2.2. Recent Advancements

It is important to remember that the presence of epithelial stem cells is necessary not only to maintain the well-being of the cornea but also to perform an effective transplant [28,30,31]. Consequently, supplying a defective cornea through a transplant in LSCD patients is impossible. As a result, stem cell therapy made it possible to help patients with LSCD, guaranteeing the permanent restoration of the transparent, self-renewing corneal epithelium.

In severe forms of LSCD, the cornea is rebuilt using a variety of LESC transplantation procedures. The International Corneal Society categorised ocular surface SC transplantation according to these criteria: source of the transplanted tissue (conjunctiva, mucosa, or keratolimbal), autologous or allogeneic (cadaver or live donor), and cell culture technique [32]. Therefore, we can distinguish the following types of limbal stem cell transplants presented in Table 1.

#### 2.2.1. CLAU

The most successful method of treating LSCD is the conjunctival-limbal autograft (CLAU), which involves transplanting a limbal fragment from a healthy contralateral eye [33,34]. The CLAU treatment involves peritomy and superficial keratectomy to remove the fibrovascular pannus from the diseased eye [35]. The damaged eye is treated by receiving two limbal transplants from the healthy contralateral eye at the hours of 12 and 6 [35]. The fact that the CLAU method needs about one-third of autologous limbal tissue from a healthy contralateral eye is a severe constraint of this surgery. This requirement can potentially induce an LSCD in the healthy eye [36,37]. A mini-CLAU, consisting of just 1–2 clock hours of limbal tissue, is an alternative option [38].

#### 2.2.2. CLET

The basis for the ex vivo cultured limbal epithelial transplantation (CLET) was presented in 1997 by Pellegrini et al. [39]. They took biopsies from the limbus of the healthy eye of two patients who suffered severe alkali burns. After a period of culture and verification of the direction of stem cell differentiation, a transplant was performed with a subsequent two-year observation. The corneas of the exposed eyes of both patients were restored with marked improvement in comfort and visual acuity [39]. Furthermore, it was confirmed that stem cells are found within the corneal limbus and contribute to the process of corneal regeneration. In 2010, Rama et al. verified long-term clinical results in 112 patients with chemical cornea burns, achieving permanent restoration of a transparent corneal epithelium in 76.6% of treated eyes [40].

Due to the promising results of auto-CLET and many patients suffering bilateral LSCD, scientists were willing to explore potential sources of cells to perform allo-CLET. In 1999, the first allo-CLET with SCs utilised from patients’ family members was performed by Schwab with prompting results for further development of technology [41]. Following that was Kinoshita’s group, which in 2001 took things one step further by performing 13 transplants utilising cadaveric donors [42]. In over 11 months of follow-up, 10 out of 13 transplanted eyes were successful [42]. Consequently, the process of ex vivo cultured limbal epithelial transplantation is subdivided into autologous and allogenic categories, respectively, according to the origin of the LESC [43]. This procedure involves taking a small number of stem cells and growing them on a scaffold. The matrix for LESC expansion may be human tissues such as the amniotic membrane, anterior lens capsules, or artificially produced materials such as silk fibroin and siloxane hydrogel [44,45]. Compared to CLAU, KLAL, and LR-CLAL, CLET is characterised by a lower risk of graft rejection, a shorter recovery time of the cornea, and a smaller number of stem cells needed to produce culture and then graft [46]. In the case of unilateral LSCD, cells used are obtained by biopsy from a healthy eye, while in bilateral, total LSCD cells are collected from a dead donor, which excludes complications in the donor [46].

#### 2.2.3. SLET

In 2012, Sangwan et al. described a new transplant technique combining the advantages of CLAU and CLET [47]. Simple limbal epithelial transplantation (SLET) needs minimum donor tissue and does not require special equipment. The procedure is based on taking a small-size sample containing the LESC, then dividing it into smaller portions and placing it on a scaffold placed on the cornea. SLET can also be performed in the auto and allogeneic variants. The benefits of SLET over CLET are considered significant since it enables cell multiplication to occur on the ocular surface instead of in a clinical laboratory [48].

#### 2.2.4. KLAL

Due to the significantly lower number of complications, no need for chronic immunosuppression, and thus better long-term treatment outcomes, autologous procedures are the options of choice. Unfortunately, without LESC, allogeneic procedures such as keratolimbal allograft (KLAL) and living-related conjunctival limbal allograft (lr-CLAL) are the methods of choice. Keratolimbal allograft involves transplanting the entire limbus extracted from a dead donor attached to the corneoscleral carrier. The described process provides a full limbus graft with a high LESC load [48,49,50]. Since KLAL involves the transplantation of richly vascularised tissue, the recipient remains under systemic immunosuppression. The most common complication of KLAL is graft rejection, but severe complications also occur, such as the transmission of donor melanoma in the transplanted limbus fragment [51].

#### 2.2.5. lr-CLAL

Living-related conjunctival allograft is a procedure technically carried out like CLAU with the difference that a graft donor is a person with the most similar HLA and AB0 antigens. Moreover, the potential donor is examined for syphilis, human immunodeficiency virus, and hepatitis B and C.

#### 2.2.6. COMET

In severe bilateral cases of LSCD, allogeneic management is associated with frequent and severe complications that significantly reduce the patient’s quality of life. The use of an autologous mucosal epithelium of non-ocular origin was therefore considered [52]. One of the therapeutic options is cultivated oral mucosal epithelial transplantation (COMET). According to the literature, this non-limbal autologous cell procedure is the most frequently used in treating bilateral LSCD [53]. To rebuild a healthy ocular surface, the process uses the autologous oral mucosal epithelium [54,55]. It is based on taking a layer of the oral mucosa, culturing it for several weeks on the amniotic membrane, and finally placing the layer on the damaged cornea. In 2015, Dobrowolski et al. performed and reported successful ocular surface reconstruction in aniridic patients using the COMET procedure. The fundamental advantage of COMET was the recovery of translucent epithelium on the ocular surface, lacking any pathologic arteries with consequent improvement in the quality of vision [56]. However, contrary to the previously mentioned study, some research groups, despite the success rate specified above 70% [56], report the occurrence of complications such as regeneration of irregular epithelial surfaces caused by peripheral neo-angiogenesis [57,58]. In addition, some ways to prevent the abovementioned complications were published [59].

#### 2.2.7. MSC

Mesenchymal stem cells are the only non-limbal allogenic stem cells successfully used in therapeutic settings. Calonge et al. reported the only clinical use of MSCs for LSCD in 2019 [60]. They compared the efficacy of MSCs graft for corneal regeneration with allo-SLET in a double-blind proof-of-concept study with a six- to twelve-month follow-up [60]. Global success at six to twelve months for CLET was between 72.7% and 77.8%, and for MSCT, between 76.5% and 85.5% [60]. The researchers found no evidence of any harmful effects caused by the cell products [60]. In addition, animal studies have demonstrated the ability to differentiate MSC into corneal epithelial cells through co-culture with rat corneal stromal cells (CSCs), which represents a significant technological advancement [60]. Moreover, researchers have found that MSCs obtained from periocular adipose tissue aspirates (AT-MSC) have the same properties as those obtained from bone marrow, thus being much more available [61]. Furthermore, AT-MSCs exert a paracrine impact by reducing trophic factor release and regulating immune response and inflammation, thereby promoting the regeneration processes [62,63]. Another promising cell source is human immature dental pulp stem cells (hIDPSCs), which display comparable essential properties as LESCs. This suggests that hIDPSCs could serve as a viable alternative for corneal restoration [64]. Additionally, it has been shown that inorganic substances increase corneal epithelial cell migration and growth [65]. Their administration in droplets may aid in ocular surface renewal [65]. 

### 2.3. Future Directions

In addition to administering stem cells to the area requiring repair, it is worthwhile to consider other indirect options of SCs therapy, such as stimulating the surrounding SCs to migrate and increase divisions for more effective treatment and, maybe in the future, regeneration of highly differentiated tissues that lack self-renewal capacity. An example of such an indirect approach representing recent discoveries suggests topical supplementation with substances that would stimulate corneal regeneration. This concept has been advanced by Li Q et al.; while still in the animal models, supplementation of MLN4924 resulted in faster corneal regeneration by remnant LSCs [66]. Alternatively, a discovery by Jang et al. offers an entirely different approach with potentially the same effect [67]. Scientists found that the increased Wnt/-β-catenin signalling in LSCD encourages the self-renewal of conjunctival stem cells and promotes corneal conjunctivalisation in animal models and human conjunctiva that has been removed from the eye [67]. Reducing or blocking this signalling could positively affect corneal transparency, leaving the LSC in patients with LSCD or pterygia more time to reconstruct a healthy cornea [67].

Despite the constantly published numerous and promising research results, before a full-scale escalation of stem cell therapy, it is necessary to better understand the processes occurring in the limbus after SCs implantation, standardisation of culturing processes, verification of effectiveness, or even LSCD diagnostics. Additionally, the following issues must be clarified: analysis of corneal epithelium after transplantation, SCs niche reconstruction process, alternative routes of SCs delivery, and other SCs sources.

## 3. Retina

### 3.1. Basic Knowledge

In human embryonic development, the retina and the optic nerve grows out from the diencephalon, which makes it the only central nervous system (CNS) element available for physical examination. The retina is made up of 10 distinct layers. The retinal pigment epithelial (RPE) cells adjacent to the vascular membrane are the most externally located. The ability of RPE cells to phagocytose photoreceptor outer segments is essential for the processes involved in the transduction of light into visual information. When either the photoreceptors or the RPE deteriorate, vision is lost. In addition, SCs have remarkable neuroprotection properties exerted by the secretion of various neurotrophic factors and immune system modulators. Thus, the direction of retinal stem cell therapy development is twofold: restoring already lost retinal cells and, secondly, the salvation of prone-to-death cells before it happens.

### 3.2. Recent Advancements

#### 3.2.1. hESC-Derived RPE Cells Replacement

Since RPE cell regeneration does not require the reconstruction of synaptic connections, progress on this approach is far more remarkable. Currently, there are 13 clinical trials registered for the transplantation of RPE cells, regardless of their stage [68]. While therapy involving RPE cells derived from pluripotent stem cells holds great promise, it faces several limitations, including poor in vivo survival and cell integration. Using cell-free ECM hydrogels with excellent biocompatibility and immunological neutrality is a possible solution to the raised issue. Guilan Li et al. reported that hydrogel obtained from porcine acellular sclera promotes graft survival and integration [69].

An increasing number of clinical trials using RPE are being published. Fernandes et al. describe a 12-month follow-up in patients with Stargardt’s disease after injecting human embryonic stem cell RPE cells (hESC-RPE). Throughout the follow-up period, the most important result was no ocular or systemic inflammation, rejection, tumor growth, or toxicity [70]. In addition, the findings of the first phase of the clinical trial involving hESC-RPE for the treatment of early-stage Stargardt macular degeneration have now been published [71]. Seven patients underwent a 5-year follow-up after subretinal transplantation of hESC-RPE cells in one eye, while the other served as a control sample. Apart from minor and transient perioperative complications in two patients (increased intraocular pressure), no other local or systemic complications were noted. Two out of seven patients at the most recent follow-up demonstrated decreased visual function from the baseline. Based on the findings, hESC-RPE can be tolerated and used safely over the long term. To obtain the best possible outcomes from treatment, the appropriate selection of patients to undergo transplantation needs supplementary development [72].

#### 3.2.2. iPSC Derived RPE Cells Replacement

Furthermore, there are initial reports on smaller clinical trials also conducted on iPSCs-induced RPE transplantation [73]. For example, Mandai et al. received iPSCs from dermal fibroblasts obtained from two patients with advanced neovascular age-related macular degeneration. The RPEs obtained as a result of iPSC differentiation have been subjected to numerous tests. One of the patients underwent a procedure involving the removal of the neovascular membrane and transplantation of the autologous iPSC-derived RPE cell sheet under the retina. At the one-year mark following surgery, the transplanted sheet had not been damaged, the patient’s best corrected visual acuity had not improved or deteriorated, and cystoid macular edema was still present [73].

#### 3.2.3. Retinal Organoids

Even though regenerative retinal neuron treatment has had less impressive results because of its many problems, it has also made a lot of progress. In addition to shared difficulties with other eye tissues, restoring effective synaptic connections between transplanted cells and host tissues remains the most difficult. In 2007, PSCs were induced for the first time from human fibroblasts [74,75]. Induced PSCs can derive 2D and 3D cell cultures, including organoids. Two-dimensional cell culture can assist in modelling neuronal development and understanding the processes behind neurological diseases. On the other hand, a three-dimensional organoid has been regarded as the gold standard for the investigation of the interaction between several types of neurons and cell organisation [76], including interactions with extracellular matrix (ECM) [77]. Brain organoids, created by Sasai’s group, were a breakthrough as they had an organised structure with several cell types that resembled the embryonic brain [78,79,80,81]. Further development of the technology by Lancaster, which was proposed by Sasai et al., opens up the prospects for creating complex structures like the retina. Retina organoids, also known as ROs, are miniature versions of the retina used to study tissue growth, maturation, and environment, as well as to stimulate the production of photoreceptors [82]. Obtaining functional ROs would greatly influence the patients diagnosed with neurodegenerative diseases affecting the retina. Numerous studies are underway to obtain organoids as close as possible to the native organ with the best survival rate and a high potential for integration with the host organism without causing rejection.

#### 3.2.4. Retinal Ganglion Cells Replacement

Optic neuropathies such as glaucoma attack afferent retinal ganglion cells (RGCs) and their axons, which compose the optic nerve together. This causes a reduction in the amount of sensory information that can be sent from the eye to the brain. Currently, the most common method used to treat glaucoma is to reduce the pressure within the eye, which does not entirely guard against the gradual deterioration of visual function. Cell replacement strategies would hold promise for millions of patients affected by glaucoma but also other neurodegenerative disorders. Over the past few years, significant progress has been made in creating RGC-like cells from embryonic stem cell origins and those originating from induced pluripotent stem cells.

Unfortunately, SCs usage in RGC replacement therapy has not yet advanced beyond the pre-clinical level [83,84]. Ex vivo integration of human stem RGCs into the mouse retina was achieved by Croteau et al. However, the success was limited due to donor cells’ poor survival in host retinas. Moreover, researchers have demonstrated the importance of supplemental support with proteins such as brain-derived neurotrophic factor (BDNF) and the adenylate cyclase activator for optimum neurite development and preservation of structural integrity. Unfortunately, there are still numerous obstacles to overcome before SCs can be used in RGC replacement therapy. The first is an ethical issue. Current cell culture methods are very expensive; using autogenous transplants, which would avoid ethical disputes, will additionally multiply the costs by preventing mass production. The second is due to the underlying reason of RGC loss and the causative genetic element; despite overcoming the expenses and producing autologous culture, we are still duplicating the defective genetic information within the recipient’s cells. Another problem is common to all regenerative retinal therapies. Attempting to restore any layer of the retina requires effective synaptogenesis. Numerous research groups have achieved promising results in animal models [85,86,87,88]. Techniques used to stimulate synaptogenesis include causing an intraocular inflammatory response [88], knocking off specific transcription factors [87], and co-manipulating the GPR17 factor [89].

#### 3.2.5. Photoreceptors Replacement

Stem cell treatments aimed at replacing already deceased photoreceptors hold the potential to cure previously untreatable conditions that lead to blindness. In 2012, Pearson and colleagues reported successful integration and restoration of visual function in mice through grafts of photoreceptor cells [90]. Unfortunately, these initially promising findings were later challenged. Multiple research groups have since demonstrated that what was initially perceived as graft integration was actually a protein exchange or fusion of cytoplasm with the recipient’s surviving photoreceptor cells [91,92]. Although further research is required to regenerate photoreceptors, the observed effect presents an alternative therapeutic approach: supplying the remaining, often impaired, photoreceptors with the necessary proteins and trophic factors to convert visual stimuli into nerve signals. Nevertheless, the recent achievement by Ripolles-Garcia et al. represents a significant milestone [93]. In this study, human photoreceptor precursor cells (PRPCs) derived in vitro were successfully transplanted into the subretinal space of seven dogs with inherited retinal degeneration. The grafts, supported by immunosuppression, lasted from 3 to 5 months and demonstrated PRPC differentiation and integration with native retinal cells [93]. Nonetheless, comprehensive research is still necessary to achieve the desired final effect.

The field of regenerative medicine is undergoing dynamic development, continually overcoming emerging limitations while simultaneously uncovering new challenges that demand attention. One such challenge is the in vivo assessment of graft cell viability, function, and signal transmission. In addition, the currently used grafts are very small in size. If there was a fusion with the host and the graft took up its function, the improvement in vision may be imperceptible. The next challenge will be to create and insert larger grafts into the eyeball while traumatising the surrounding tissues as little as possible. Combining ROs transplants with gene therapy may also be a beneficial direction for the patient [94].

A recent study on the development of SCs into photoreceptor progenitor cells was published [95]. Scientists generated a human recombinant retina-specific laminin isoform and demonstrated its role in promoting the differentiation of human embryonic stem cells into photoreceptor progenitors in just 32 days. The progenitor cells collected were examined in mouse and rabbit models. During the over-20-week-long follow-up, the transplanted cells’ viability and the formation of new synaptic connections were demonstrated. In addition, researchers reported no teratoma growth, along with partial improvement in vision [95]. Reproducing the results of animal models in humans would create a chance for the safe management of retinal degenerative diseases.

Additionally, a clinical trial investigating the potential use of retinal stem and progenitor cells in treating AMD started in 2022 [96]. The project goal is to create a biomedical cell product based on cells from the retinal pigment epithelium that will be highly successful in treating age-related macular degeneration. The increased interest in the subject gives rise to optimism for further advancement in applying these cells, even though the study’s findings are not yet available.

Recently, the role of Muller’s glial cells (MG) in maintaining the well-being of the retina has been explored [97,98,99]. MG cells are an element of highly differentiated tissue. MG reprograms itself to acquire stem cell characteristics in response to retinal damage. This reprogramming causes nuclear migration and asymmetric cell division. Asymmetric cell division produces a temporarily proliferating multipotent progenitor that restores the original Müller glia. In addition, multipotent progenitors migrate to all cell layers, exit the cell cycle, and regenerate the main retinal cell types [100]. In 2008, Karl et al. showed in mice that the mammalian retina has the potential to regenerate inner retinal neurons in vivo [97]. The mice tested had amacrine cells and ganglion cells removed by NMDA injections. Then, using trophic factors, MG cells in mice’s retina were put into ”healing mode”, and the regeneration of previously destroyed cells was assessed. After initial findings, numerous animal models have shown that MG cells cultured with certain trophic factors are induced into a regenerative state [97]. Nowadays, it is known that MG cells found in amphibians, fish, birds, and even some mammals in specific conditions, have strong regenerative abilities, resulting in, for example, the reconstruction of a damaged or even destroyed retina [97,101,102,103,104]. New therapies for retinal degeneration may result from further research into possible limits of MG cells induction. The future of retinal regeneration may lie in the combination of ROs and MG to produce numerous and effective synaptic connections.

### 3.3. Future Directions

Further advancements in the use of SCs may prevent glaucomatous neuropathy’s progression, and regenerating retinal ganglion cells may result in the patient not losing sight or having a significantly narrowed field view. Replacing damaged retinal photoreceptors and RPE in inherited retinal degeneration gives patients a chance to maintain visual acuity and visual field. Regeneration of RPE cells in treating dry and wet AMD would preserve central vision (so-called macular vision), which is completely abolished in advanced AMD. In such a condition, the patient sees the central patch based only on weaker and less accurate peripheral vision.

## 4. Conjunctiva

### 4.1. Basic Knowledge

The thin mucous membrane known as the conjunctiva borders the interior of the eyelids and protects the sclera from foreign objects. Typically, the conjunctiva is separated into three sections: palpebral, bulbar, and fornix. The nonkeratinised, stratified squamous and columnar epithelium found throughout the tissue makes up the conjunctiva. Dispersed goblet cells are also present. It functions as a mechanical barrier but also protects against pathogens via antimicrobial peptides and conjunctiva-associated lymphoid tissue (CALT). Additionally, it prevents fluid loss through the secretion of mucin. The localised illness can cause the thickness of the conjunctiva, forniceal shortening, symblepharon, dry eye syndrome, and subsequent corneal opacities.

The conjunctival stem cells (CjSC) may be found throughout the human conjunctival epithelium; however, the regions of the medial canthal and inferior forniceal have much higher densities of these cells [105]. It has also been shown that cells collected faster after death and from younger donors have a greater potential to create cell-rich grafts [106]. In addition, it is believed that conjunctival keratinocytes and mucin-producing goblet cells originate from a single progenitor, which is an essential finding in developing grafts [105].

Ocular conditions that alter the cornea also impact the conjunctiva. Hence, treating both neighbouring tissues is sometimes necessary. The transplantation of the amniotic membrane, oral mucosa, nasal turbinate mucosa, and autologous or allogeneic conjunctiva are some of the approaches that are now being employed effectively [107]. These methods, however, have their limits in terms of total regeneration. Additionally, the availability of autogenic or allogenic tissue is quite limited. Nevertheless, the use of hydrogel scaffolds for the curative transport of SCs to the ocular surface has made some positive advances over the past few years [108,109]. Further, initial reports have emerged regarding the successful application of bioprinted injectable hydrogel micro-constructs loaded with CjSCs for the treatment of severe surface disorders in animals [110]. 

### 4.2. Future Directions

The successful usage of SCs in conjunctival diseases allows one to maintain the conjunctiva’s continuity and properly create an external mechanical barrier. Furthermore, conjunctival regeneration causes the mucin component of the tear film to be secreted correctly by conjunctival goblet cells. Thanks to this, there is no disturbance in the composition of the tear film leading to the symptoms of dry eye syndrome in the patient, which in the advanced stage may cause blurred vision and decreased visual acuity.

## 5. Iris

### Basic Knowledge and Future Directions

Iris is a ring-shaped structure that controls the quantity of light that reaches the eye’s retina through its contraction. Its surface divides the globe into anterior and posterior chambers. It is part of the middle vascular layer of the eye—the other two components are the choroid and the ciliary body.

Under certain circumstances, iris pigment epithelial (IPE) cells were proven to differentiate into retinal photoreceptor-like cells and retinal ganglion cells. Moreover, IPE cells can secrete essential neurotrophins and bind toxic iron ions, which constitute initial events causing the loss of cells in Parkinson’s disease. Before IPE cells may be used in clinical settings, additional research must be conducted on the topic [111], but it seems to be a good place to source SC from.

## 6. Trabecular Meshwork

### Basic Knowledge and Future Directions

The trabecular meshwork (TM) is located at the angle formed by the cornea and the iris in the anterior part of the eye. It is accountable for the outflow of aqueous fluid. Together with the aqueous secretion, it has a crucial impact on intraocular pressure (IOP), which is a significant contributor to the development of glaucoma. As a result, there has been a notable increase in research dedicated to understanding TM cells and their distinctive characteristics. Numerous studies show that TM cells can differentiate into adipocytes, chondrocytes, and osteocytes [112]. In addition to this, it has been demonstrated to clear debris from the circulating aqueous humor [113].

While treating glaucoma, the intraocular pressure is still the primary focus of treatment, whereas the trabecular meshwork is the primary location of outflow resistance. It has been suggested that a decrease in TM cells and abnormal ECM are linked to increased resistance to outflow [114]. TM stem cells (TMSC) are abundant in the TM’s insert area and are characterised as capable of regenerating TM cells. Certain animal models, which reflect primary open-angle glaucoma (POAG) phenotypes, are available [114]. However, the effectiveness of TMSCs in TM regeneration and its function is yet to be established. Hopefully, using SCs to regenerate the trabecular meshwork could be a treatment option for glaucoma, ultimately leading to optic nerve damage and blindness.

## 7. Ciliary Body

### Basic Knowledge and Future Directions

The ciliary body is a part of the eye consisting of three important elements: the ciliary muscle responsible for changing the shape of the lens, ciliary processes, and pars plana, both covered by epithelium producing the aqueous humor. It also plays a role in keeping the eye’s immune-privileged state intact.

Recent research has demonstrated that some of the cells once thought to be retinal stem cells are pigmented ciliary epithelial cells (CEC), suggesting that CEC can differentiate into the retinal lineage [115]. Additionally, researchers know how to bring the CEC into a pluripotent state [116]. However, according to in vivo observations, CECs differentiated in retinal lineage do not integrate with existing retinal architecture. Additionally, it has been claimed that the cells that make up the ciliary epithelium cannot develop into photoreceptors [117,118]. The exact nature of these cells is still unknown; therefore, we need more research to use CEC in regenerative medicine. It may be possible to use ciliary body cells to regenerate damaged retina.

## 8. Lens

### Basic Knowledge and Future Directions

The lens comprises three main components: the capsule, the epithelium, and the fibres. It is a transparent, elastic, and biconvex structure. Its refractive power is around 20 diopters, making it one-third of the eye’s total refractive power. Through changing its shape, it shifts focal length so that we can focus on objects at various distances.

Currently, the standard cataract treatment involves removing and replacing the natural lens with a synthetic one. While often utilised in cataract surgery, artificial intraocular lenses are hindered by dislocation, improper lens power, artificial lens calcification, and optical defects [119]. The latest advancement in stem cell therapy for lens treatment involves utilising residual lens epithelial SCs to regenerate a healthy lens following the removal of a diseased lens [120]. The damaged lens is removed during this procedure, leaving the lens epithelial stem cells intact. The latter rebuild the lens within six months. Lin et al. successfully performed 12 such treatments on infants under two years of age [120]. A different strategy is based on the rapid development of ROs and its focus on making lens organoids or even primitive eyes with a retina, a lens, and a cornea [121,122].

Replacing the cataract-changed lens (impairing the patient’s vision) with a new transparent lens would be a revolutionary solution, especially in children with congenital cataracts, in whom removal of the cataractous lens in infancy leaves the eye without a lens (usually for several years). This involves using glasses or contact lenses with very high power to replace the breaking force of the natural lens. In addition, the reconstructed lens could still have accommodative abilities, which artificial intraocular lenses do not have.

## 9. Sclera

### Basic Knowledge and Future Directions

Surgical treatment for myopia includes laser refractive corneal surgery, phakic posterior chamber intraocular lens implantation, and posterior scleral reinforcement (PSR). In the past few years, researchers have also suggested injecting dopamine and subdural mesenchymal SCs to treat high myopia, which is a potential new method to arrest the growth of myopia [123].

Myopia poses a significant socioeconomic concern, primarily due to the increasing number of individuals affected by this condition worldwide and its progressive nature throughout a person’s life (axial lengthening of the eyeball). Moreover, advanced degenerative myopia can lead to various impairments in the eye’s fundus caused by extensive stretching and thinning of tissues, necessitating the use of high-power lenses. Unfortunately, this increases the risk of severe vision damage, including retinal detachment or alterations in the macular region, which is responsible for the clearest, central vision. The ability to use SCs to stop the progression of myopia would indeed be a groundbreaking breakthrough.

## 10. Orbital Fat

### Basic Knowledge and Future Directions

The orbit serves as the eye’s framework. It contains the eye, optic nerve, oculomotor muscles, adipose tissue, lacrimal gland, and blood vessels. The structure of the eyeball is complex, and it has numerous connections with other structures. The proximity to the cranial vault and nasal cavity is a potential way of spreading disease processes such as infections, inflammations, and cancer.

Current research has extracted and described stem cells originating from orbital fat (OFSC). It has been demonstrated that they can develop into osteoblasts, chondrocytes, and adipocytes [122]. Moreover, when cultured with corneal epithelial cells, OFSC changes their phenotypic expression to be similar to that represented by epithelial cells. Hence topical administration of OFSCs in cornea regeneration has been developed [124], which may improve SC therapy in corneal diseases.

## 11. Conclusions

Ophthalmology is a leading branch of regenerative medicine, rapidly developing in using SCs. SCs are already widely used in corneal transplants, whereas numerous techniques of LESC transplantation are described. Additionally, RPE cell transplants are the basis of many ongoing clinical trials. The use of SCs in the conjunctiva, iris, trabecular meshwork, lens, ciliary body, sclera, and orbital fat is being intensively studied. Here we present the latest data on the stem cells of individual tissues and their applications, aware that with the constant rate of development, it can soon require updating. Following the progress in the use of SCs in ophthalmology, it must be borne in mind that depending on the eye tissue, the quantity and quality of scientific research varies significantly. In addition, numerous clinical trials are underway on the above topic, the results of which are not yet known. Regular reports on the proven applications of stem cells will certainly sooner or later change the patterns of conduct in today’s ophthalmology.

## Figures and Tables

**Table 1:** Classification of limbal epithelial stem cell transplants. The most common LESC transplantation techniques by stem cell source.

Limbal Autografts	Limbal Allografts	Non-LESCs Transplantation
Conjunctival-limbal autograft (CLAU)	Keratolimbal allografts (KLAL)	Cultivated oral mucosal epithelial transplantation (COMET)
Cultured limbal epithelial transplantation (CLET)	Living-related conjunctival allograft (LR-CLAL)	
Simple limbal epithelial transplantation (SLET)	Allogenic SLET	

## Data Availability

No new data were created or analyzed in this study. Data sharing is not applicable to this article.

## Supplementary Materials

The following supporting information can be downloaded at: https://www.mdpi.com/article/10.3390/biology12050737/s1. Table S1: Summary of the nine clinical trials in retinal diseases.
