# Supplementary material for: Advancements in Ocular Regenerative Therapies
Source: Biology (Basel). 2023 May 19;12(5):737. doi: 10.3390/biology12050737 (PMC10215726; doi:10.3390/biology12050737)
Supplement: Supplementary file 1 [file biology-12-00737-s001.zip › biology-2360790-supplementary.pdf]

*Review*

# Advancements in Ocular Regenerative Therapies

**Wojciech Tomczak, Weronika Winkler-Lach, Martyna Tomczyk-Socha and Marta Misiuk-Hojło**

**Table S1.** Summary of the nine clinical trials in retinal diseases.

| Title | NCT number | Status | Design | Brief description | Age/group demografics | Conditions |
|---|---|---|---|---|---|---|
| **Bone Marrow Derived Stem Cell Ophthalmology Treatment Study II** | NCT03011541 | Recruiting | • Intervention Model: Single Group Assignment<br>• Intervention Model Description: Single Arm- Arm 1. Comparator is natural history of the disease.<br>• Masking: None (Open Label)<br>• Primary Purpose: Treatment | Evaluating the use of autologous bone marrow-derived stem cells (BMSC) for the treatment of retinal and optic nerve damage or disease | 18 years and older | • Retinal Disease<br>• Age-Related Macular Degeneration<br>• Retinitis Pigmentosa<br>• Stargardt Disease<br>• Optic Neuropathy<br>• Nonarteritic Ischemic Optic Neuropathy<br>• Optic Atrophy<br>• Optic Nerve Disease<br>• Glaucoma<br>• Leber Hereditary Optic Neuropathy<br>• Blindness<br>• Vision Loss Night<br>• Vision Loss Partial<br>• Vision, Low<br>• Retinopathy<br>• Maculopathy<br>• Macular Degeneration<br>• Retina Atrophy |
| **Follow-up Study After ACLSCT for Restoration of Corneal Epithelium in** | NCT03288844 | Active, not recruiting | • Multinational<br>• Multicentre | A follow-up study of patients transplanted in the HOLOCORE clinical trial who consent to participate. | Adults and children | • Limbal Stem Cell Deficiency Due to Ocular Burn |

| | | | | | | |
|---|---|---|---|---|---|---|
| **Patients With LSCD Due to Ocular Burns** | | | • Time perspective: Prospective<br>• Primary Purpose: to collect long-term efficacy and safety data | | | |
| **Cultured Autologous Oral Mucosa Epithelial Sheet for the Treatment of Bilateral Limbal Stem Cell Deficiency** | NCT03949881 | Recruiting | • Intervention Model: Single Group Assignment<br>• Masking: None (Open Label)<br>• Primary Purpose: Treatment | A clinical trial performed to evaluate tolerance and efficacy of the autologous jugal mucosa cell sheet cultured using innovative process. | 18 years and older | • Total Bilateral Limbal Cell Deficiency |
| **Treatment of Central Retinal Vein Occlusion Using Stem Cells Study** | NCT03981549 | Active, not recruiting | • Allocation: Randomized<br>• Intervention Model: Parallel Assignment<br>• Masking: Triple (Participant, Investigator, Outcomes Assessor)<br>• Primary Purpose: Treatment | To evaluate whether intravitreal autologous CD34+ stem cell therapy is safe, feasible and potentially beneficial in eyes with vision loss from central retinal vein occlusion (CRVO). | 18 years and older | • Central Retinal Vein Occlusion |
| **The Effects of Allogeneic Simple Limbal Epithelial Transplantation** | NCT04021134 | Recruiting | • Observational Model: Case-Only<br>• Time Perspective: Prospective<br>• Primary Purpose: assessing the SLET effectiveness | To investigate the effect of allogeneic SLET and re-epithelialization after allogeneic SLET. | 10 to 90 years old | • Limbal Stem-cell Deficiency |
| **Efficacy of Locally Delivered Allogeneic Mesenchymal Stromal Cells** | NCT05705024 | Not yet recruiting | • Allocation: Randomized<br>• Intervention Model: Parallel Assignment<br>• Masking: Double (Participant, Care Provider)<br>• Primary Purpose: Treatment | The assessment of efficacy of locally delivered allogenic mesenchymal stromal cells on chronic epitheliopathies. | 18 years and older | • Corneal Ulcer |

| | | | | | | |
|---|---|---|---|---|---|---|
| **Treatment With Allogeneic Adipose-derived Mesenchymal Stem Cells in Patients With Aqueous Deficient Dry Eye Disease** | NCT03878628 | Active, not recruiting | • Intervention Model: Single Group Assignment<br>• Masking: None (Open Label)<br>• Primary Purpose: Treatment | To assess the safety and feasibility of allogeneic adipose tissue-derived mesenchymal stem cells injected into the lacrimal gland in patients with Aqueous Deficient Dry Eye Disease. | 18 years and older | • Dry Eye<br>• Kerato Conjunctivitis Sicca<br>• Aqueous Tear Deficiency |
| **Ocular Graft-Versus-Host-Disease After Allogeneic Haematopoietic Stem Cell Transplantation** | NCT05170347 | Recruiting | • Observational Model: Cohort<br>• Time Perspective: Prospective<br>• Primary Purpose: to collect data end expand knowledge | Ocular GVHD occurs in 30-70% of patients after HSCT. oGVHD can severely impact patients' quality of life. This study aims to understand the population-based epidemiology of oGVHD and long-term ophthalmic outcomes. | 18 years and older | • Graft Vs Host Disease<br>• Haematological Malignancy<br>• Cancer |
| **A Safety Surveillance Study in Subjects with Macular Degenerative Disease Treated With Human Embryonic Stem Cell-derived Retinal Pigment Epithelial Cell Therapy** | NCT03167203 | Enrolling by invitation | • Intervention Model: Single Group Assignment<br>• Masking: None (Open Label)<br>• Primary Purpose: Treatment | To evaluate late-onset adverse events in AIRM-sponsored clinical trial participants who underwent sub-retinal transplants of human embryonic stem cell-derived retinal pigment epithelial (hESC-RPE) cells. | 18 years and older | • Macular Degenerative Disease |
